# MEA-Net: multilayer edge attention network for medical image segmentation

**DOI:** 10.1038/s41598-022-11852-y

**Published:** 2022-05-12

**Authors:** Huilin Liu, Yue Feng, Hong Xu, Shufen Liang, Huizhu Liang, Shengke Li, Jiajian Zhu, Shuai Yang, Fufeng Li

**Affiliations:** 1 Faculty of Intelligent Manufacturing, Wuyi University, Jiangmen, Guangdong China; 2 Victoria University, Melbourne, Australia; 3 Laboratory of TCM Four Processing, Shanghai University of TCM, Shanghai, China

**Keywords:** Computational biology and bioinformatics, Medical research

## Abstract

Medical image segmentation is a fundamental step in medical analysis and diagnosis. In recent years, deep learning networks have been used for precise segmentation. Numerous improved encoder–decoder structures have been proposed for various segmentation tasks. However, high-level features have gained more research attention than the abundant low-level features in the early stages of segmentation. Consequently, the learning of edge feature maps has been limited, which can lead to ambiguous boundaries of the predicted results. Inspired by the encoder–decoder network and attention mechanism, this study investigates a novel multilayer edge attention network (MEA-Net) to fully utilize the edge information in the encoding stages. MEA-Net comprises three major components: a feature encoder module, a feature decoder module, and an edge module. An edge feature extraction module in the edge module is designed to produce edge feature maps by a sequence of convolution operations so as to integrate the inconsistent edge information from different encoding stages. A multilayer attention guidance module is designed to use each attention feature map to filter edge information and select important and useful features. Through experiments, MEA-Net is evaluated on four medical image datasets, including tongue images, retinal vessel images, lung images, and clinical images. The evaluation values of the Accuracy of four medical image datasets are 0.9957, 0.9736, 0.9942, and 0.9993, respectively. The values of the Dice coefficient are 0.9902, 0.8377, 0.9885, and 0.9704, respectively. Experimental results demonstrate that the network being studied outperforms current state-of-the-art methods in terms of the five commonly used evaluation metrics. The proposed MEA-Net can be used for the early diagnosis of relevant diseases. In addition, clinicians can obtain more accurate clinical information from segmented medical images.

## Introduction

Medical image segmentation is a key step in medical image applications. With the development of image processing techniques and machine learning methods, several state-of-the-art deep learning (DL) algorithms have been applied to medical image segmentation owing to their excellent feature extraction capability^1–5^. To obtain a segmentation model with high accuracy, DL-based models need to be trained with a significant amount of image data. However, it is difficult to obtain a tremendous amount of annotated image data because clinical experts annotate a large number of segmentation masks with pixels, which is an expensive and time-consuming process^6^.

Hence, U-Net^1^ has been proposed for biomedical image segmentation because it requires only a small number of training samples and is commonly used in medical image analysis. Many variations based on the encoder–decoder structure have been proposed for different medical image segmentation tasks^7–10^. DENSE-Inception U-Net^11^ integrates the Inception-Res module^12,13^, densely connecting the convolutional modules for extraction of features and deepening of the network without additional parameters. CE-Net^14^ applies different receptive fields to detect different sizes of targets, obtaining more high-level feature information in medical imaging.

On the other hand, many researchers have introduced attention mechanisms to obtain necessary information^15^. Attention U-Net^16^ uses a novel attention gate module to highlight salient features between the encoding and decoding paths. GC-Net^17^ designs global context attention in the decoding path to produce more representative features. CPFNet^18^ proposes multiple global pyramid guidance to obtain different levels of global context information in a skip connection.

However, the aforementioned systems only use deep image features for segmentation, ignoring shallow image features^19^. Although DL has been successfully used to improve the performance of medical image segmentation, the capability to suppress redundant information is still limited.

The deep layers of U-Net provide a high-level feature map with rich semantic information, and its shallow layers provide a low-level detailed feature map, such as edge, color, and gradients^20^. With the development of U-Net variants, it is evident that rich low-level features are critical in medical image segmentation. Researchers have increasingly studied the influence of edge information on the performance of medical image segmentation^21–23^.

To effectively use edge information, one of the low-level features, several new networks have been proposed to predict medical image segmentation. Shallow layers in the encoding path have richer detailed information and less semantic information. In contrast, deeper layers with large receptive fields have abundant semantic information but lack detailed information. TongueNet^21^ developed a morphological processing layer to detect the edges and refine the predicted results. Holistically-nested edge detection^22^ focuses on rich hierarchical representations to resolve the challenging ambiguity in edge and object boundary detection. To capture richer convolutional features, the edge detection module^24^ fully exploits multi-scale and multi-level information for edge detection, achieving remarkable performance. ET-Net^25^ designed an edge guidance module with an attention mechanism in the early stage such that it utilizes edge information to monitor and guide the segmentation process. AEC-Net^26^ introduced an attention mechanism to learn edge and texture features simultaneously in the encoding path.

**Figure 1:**
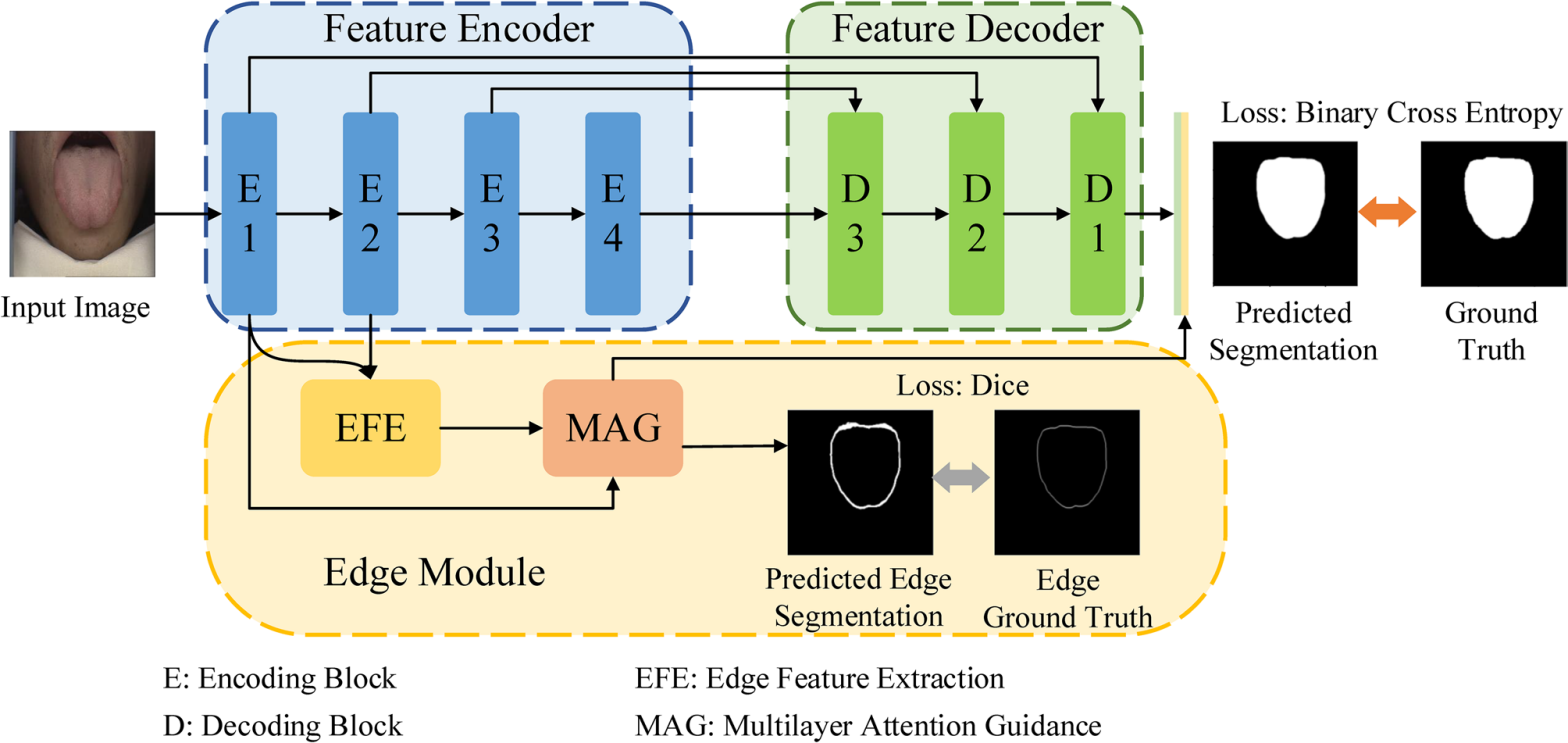
Overview of the MEA-Net (a feature encoder, a feature decoder, and an edge module).

Motivated by the functional gaps in current attention mechanism systems, we propose a novel multilayer edge attention network (MEA-Net), as shown in Fig. 1. The network comprises two new blocks in the edge module: edge feature extraction (EFE) and multilayer attention guidance (MAG). EFE produces new edge feature maps in the early stages, and the MAG combines different individual feature maps with an attention mechanism to screen more abundant edge information.

This study demonstrates three aspects as follows:

1. The EFE module captures and preserves edge information in the early encoding path.
2. The MAG module suppresses irrelevant information and chooses discriminative and effective features.
3. Experiments conducted on three publicly available datasets and one clinical image dataset, results indicate that MEA-Net performs well for different segmentation tasks.

## Methods

### Overview

The architecture of the proposed network is illustrated in Fig. 1. The proposed MEA-Net consists of three main parts: a feature encoder, a feature decoder, and an edge module. The feature encoder employs a sequence of convolution and down-sampling to extract various feature maps. The feature decoder is composed of three cascaded decoding blocks, which are used to concatenate features from the encoding and decoding paths. The edge module contains the EFE and MAG modules. The EFE module is used to capture edge information and produce edge attention maps in the early stages. The MAG module is used to filter edge information with different attention maps and obtain representative feature maps. Finally, the predicted map and edge map are combined, and then a convolution operation is performed to achieve the best prediction.

### Feature encoder

**Figure 2:**
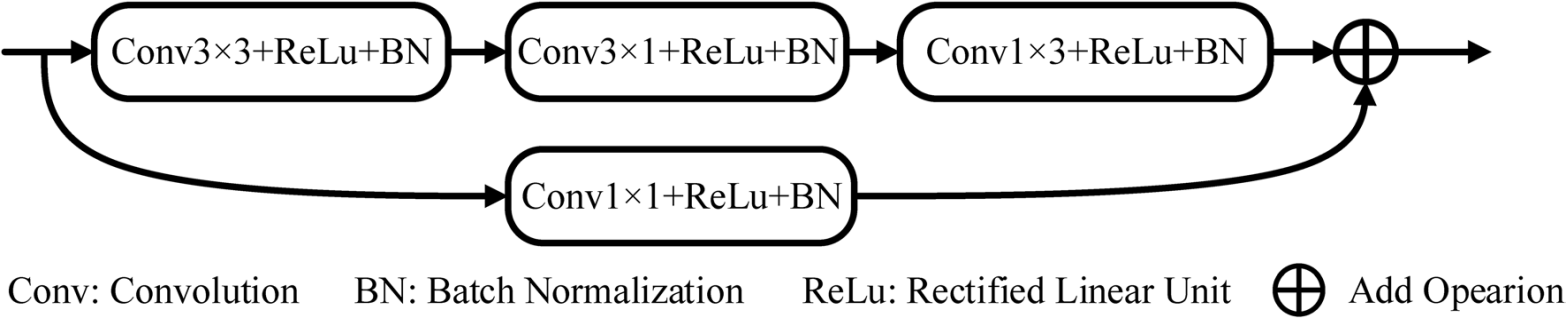
Encoding block.

The encoder modules in encoder–decoder networks^14,18,27,28^ typically use ResNet as the pretraining model. However, the pretraining model is trained by datasets such as Cityscape^29^ and ImageNet^30^, which are used in semantic scene segmentation^17^. It is unsuitable for medical image segmentation. Therefore, we have designed a new feature encoder to extract more information as shown in Fig. 2. To extract local information, a simple 3 × 3 convolution with a rectified linear unit (ReLU) and a batch normalization (BN) is used at the beginning of each feature encoder to enlarge the receptive field and allow for the capturing of more complex features. Following the 3 × 3 convolution module, two asymmetric convolutions^31,32^ (3 × 1 and 1 × 3) with ReLU and BN are used to reduce computational complexity. We have also added a residual connection of the 1 × 1 convolutional layer including ReLU and BN to obtain some additional spatial information in medical image segmentation.

### Feature decoder

**Figure 3:**
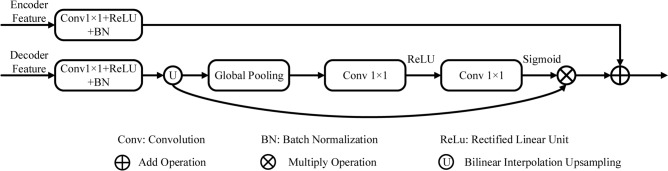
Decoding block.

To restore high-resolution feature maps efficiently and better save useful information, new decoder blocks are used in the decoder path. In Ref 1, feature maps from the decoding path are only linked to the correspondingly copied feature maps from the encoding path, so a semantic gap between the two sets of features emerges. Therefore, we have designed a new feature decoder to bridge the gap and fuse the feature maps from different paths as shown in Fig. 3. Motivated by the skip connection and attention mechanism, the feature decoder includes two branches. In the first branch, low-level features undergo a 1 × 1 convolution to generate detailed information features. In the second branch, high-level features undergo a 1 × 1 convolution to produce new features that are restored to the same size as low-level features by bilinear interpolation. Then, these new features undergo global max pooling to realize the global context features. Then, two 1 × 1 convolutional layers with different non-linearity activation functions (i.e., ReLU and Sigmoid) are used to generate the relevant weights. Next, these new features are multiplied by these weights to obtain the global features. Finally, the global features are combined with the output of the first branch to produce more representative feature maps in the decoding path.

### Edge module

**Figure 4:**
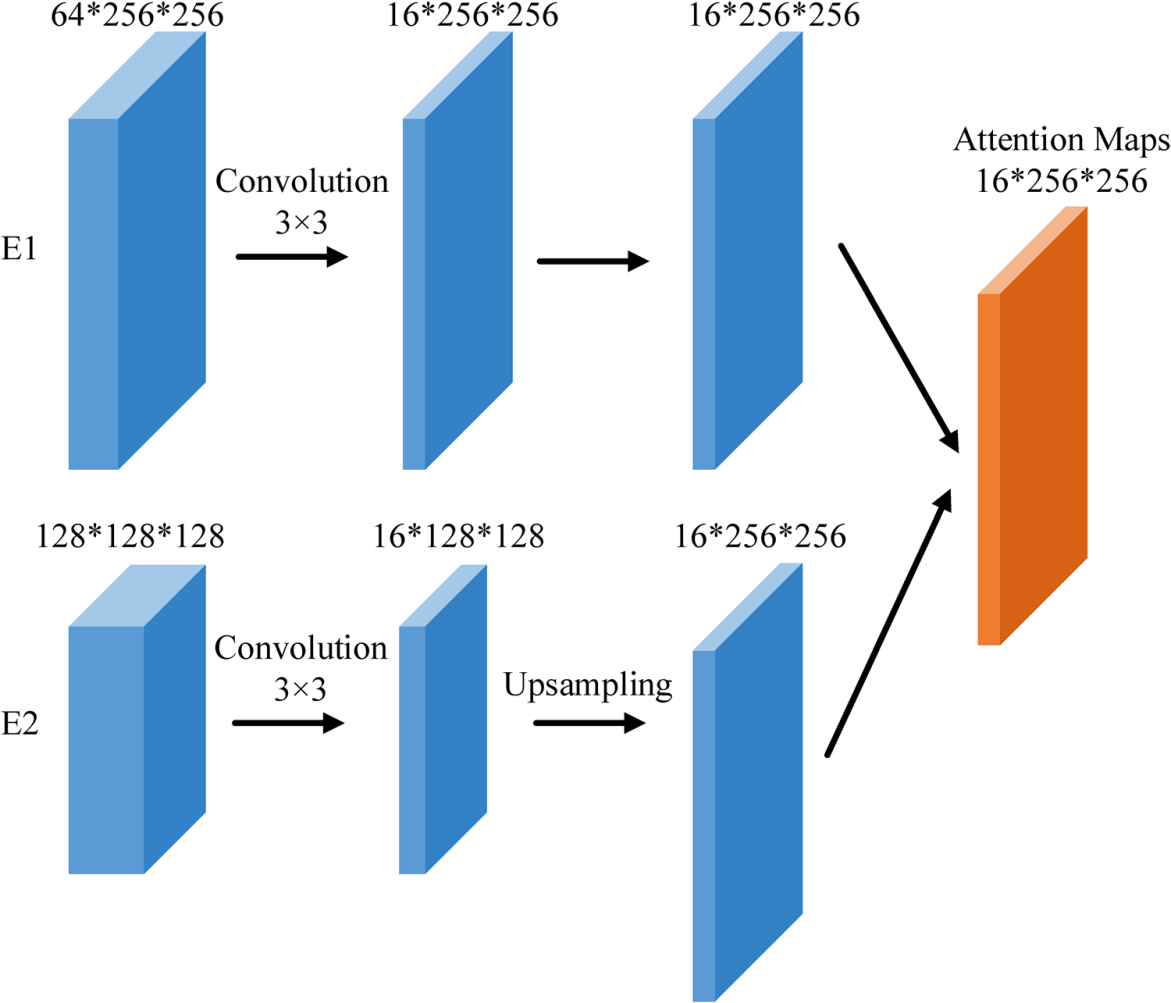
Edge feature extraction.

**Figure 5:**
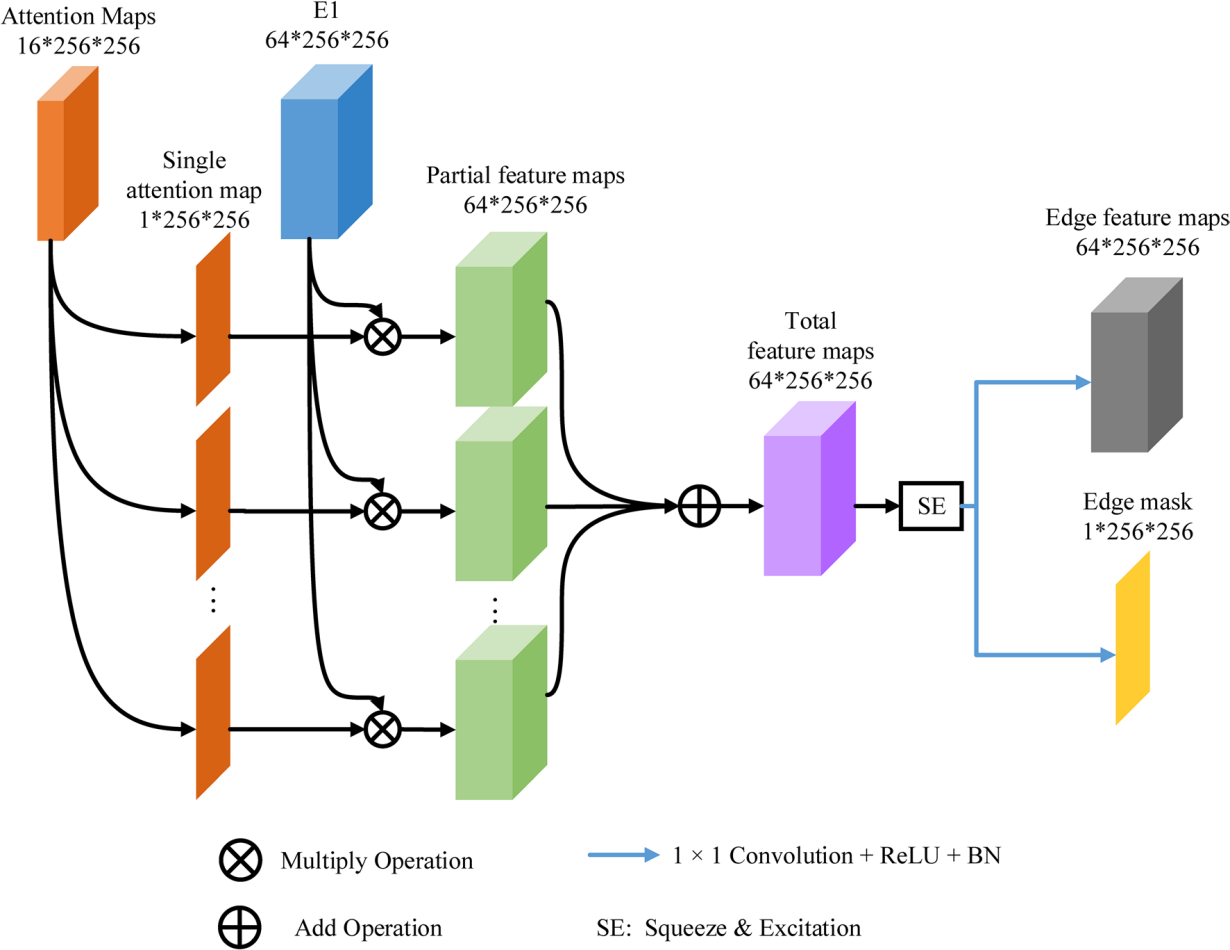
Multilayer attention guidance.

Low-level features in the early stages preserve sufficient edge information. Low-level information may be progressively weakened when it is gradually transmitted to deeper layers^33^. To make good use of this edge information, we have designed the EFE and MAG modules, as shown in Figs. 4 and 5.

#### EFE

The receptive fields of the feature maps in the Encoding Block1 (E1) and the Encoding Block2 (E2) are different. Therefore, directly combining them can result in unsatisfactory results. Inspired by this problem, the developed EFE module (Fig. 4) can provide ample edge attention maps and preserve local edge characteristics in the early stages. First, the features of both E1 and E2 are mapped into 16 channels by a 3 × 3 convolution. Next, the generated feature maps from E2 are upsampled to the same resolution as E1. The two new feature maps are combined to capture and produce edge attention maps. The number of attention maps is 16, so we can obtain 16 different attention maps with various edge information. The EFE module can be summarized as follows:

1 $${\mathbf{A}} = Conv_{3 \times 3} \left( {{\mathbf{X}}_{1} } \right) + U\left[ {Conv_{3 \times 3} \left( {{\mathbf{X}}_{2} } \right)} \right]$$

where ${\mathbf{A}}$ donates the output of the EFE module in the edge module, ${\mathbf{X}}_{1}$ and ${\mathbf{X}}_{2}$ are the inputs of EFEproduced from E1 and the E2 respectively, $Conv_{3 \times 3} ( \cdot )$ represents the 3 × 3 convolution operation followed by one ReLU and one batch normalization, and $U\left[ \cdot \right]$ denotes a bilinear interpolation upsampling with a rate of 2.

#### MAG

As discussed in the introduction, a large amount of edge information in the early stages can refine the spatial information of high-level features and restore image details. Motivated by the attention pooling module^34^ which associates attention outputs and feature maps, the MAG module (Fig. 5) is proposed to filter edge information and choose discriminative and effective features. The multilayer attention maps produced by the EFE module have different channel information. Each attention map ${\mathbf{A}}_{1} ,...,{\mathbf{A}}_{m}$ is multiplied by ${\mathbf{X}}_{1}$ to produce new features ${\mathbf{U}}_{part}$ with an attention bias. Then, partial feature maps ${\mathbf{U}}_{part}$ are summed to form the total feature maps ${\mathbf{U}}_{total}$.

2 $${\mathbf{U}}_{total} = \sum\limits_{m = 1}^{N} {\left( {{\mathbf{U}}_{{part_{{\text{m}}} }} } \right)} { = }\sum\limits_{m = 1}^{N} {\left( {{\mathbf{A}}_{m} \otimes {\mathbf{X}}_{1} } \right) \, } \left( {N = 16} \right)$$

After that, these new features ${\mathbf{U}}_{total}$ go through a squeeze & excitation (SE) block^35^ to improve the ability to extract the global edge features. First, the feature maps ${\mathbf{U}}_{total} { = }\left[ {{\mathbf{u}}_{1} ,{\mathbf{u}}_{2} , \cdot \cdot \cdot ,{\mathbf{u}}_{C} } \right]$ are considered a combination of channels ${\mathbf{u}}_{i} \in {\mathbb{R}}^{H \times W}$, performing spatial squeeze by a global average pooling layer and producing a vector ${\mathbf{z}} \in {\mathbb{R}}^{1 \times 1 \times C}$ with its $k{th}$ element:

3 $$z_{k} = F_{squeeze} ({\mathbf{u}}) = \frac{1}{H \times W}\sum\limits_{i}^{H} {\sum\limits_{j}^{W} {{\mathbf{u}}_{k} \left( {i,j} \right)} }$$

where $\left( {i,j} \right)$ is the location of the input feature maps, H and W represent the spatial height and width.

Then, to make full use of the edge information aggregated in the squeeze operation, the excitation operation is used to capture channel-wise dependencies by a simple gating mechanism with a sigmoid activation ^35^

4 $${\mathbf{s}} = F_{excitation} ({\mathbf{z}}) = \sigma \left( {{\tilde{\mathbf{z}}}} \right){ = }\sigma \left( {{\mathbf{W}}_{1} \left( {\delta \left( {{\mathbf{W}}_{2} {\mathbf{z}}} \right)} \right)} \right)$$

where ${\mathbf{W}}_{1} \in {\mathbb{R}}^{{C \times \frac{C}{16}}}$ and ${\mathbf{W}}_{2} \in {\mathbb{R}}^{{\frac{C}{16} \times C}}$ refer to the weight of two fully connected layers respectively. $\delta \left( \cdot \right)$ denotes the ReLU function and $\sigma \left( \cdot \right)$ is a sigmoid layer to reset the value of the activations of ${\tilde{\mathbf{z}}}$ between the interval [0,1].

These activations are adaptively tuned to ignore unnecessary channels and emphasize the important ones. The final output of the block is obtained by rescaling ${\mathbf{U}}_{total}$ with the activations **s:**

5 $${\tilde{\mathbf{U}}} = {\mathbf{F}}_{SE} ({\mathbf{U}}_{total} ) = {\mathbf{F}}_{scale} ({\mathbf{U}}_{total} ,{\mathbf{s}}) = \left[ {s_{1} u_{1} ,s_{2} u_{2} , \cdot \cdot \cdot ,s_{C} u_{C} } \right]$$

Finally, the feature maps ${\tilde{\mathbf{U}}}$ pass through one of two branches: a 1 × 1 convolution operation to produce the edge features ${\mathbf{Y}}_{1}$ in the decoding path, and another 1 × 1 convolution operation to predict the edge segmentation ${\mathbf{Y}}_{2}$ for early supervision.

6 $${\mathbf{Y}}_{1} = Conv_{1 \times 1} \left( {{\tilde{\mathbf{U}}}} \right)$$

7 $${\mathbf{Y}}_{2} = Conv_{1 \times 1} \left( {{\tilde{\mathbf{U}}}} \right)$$

where $Conv_{1 \times 1} \left( \cdot \right)$ represents the 1 × 1 convolution operation, followed by one ReLU activation and one batch normalization.

### Loss function

The loss function for medical image segmentation typically considers class distribution imbalance. In our experiment, the tongue region is larger than the retinal vessel region in the image. To adapt the characteristics of different datasets, the Dice loss^36,37^ is used in the edge module, whereas the binary cross-entropy loss function^38^ is employed in the final segmentation results. These two functions’ formulas are as follows:

8 $$L_{Dice} = 1 - \frac{{2\sum\limits_{i}^{N} {p\left( {k,i} \right)g\left( {k,i} \right)} }}{{\sum\limits_{i}^{N} {p^{2} \left( {k,i} \right)} + \sum\limits_{i}^{N} {g^{2} \left( {k,i} \right)} }}$$

9 $$L_{BCE} = - g\left( {k,i} \right)\log \left[ {p\left( {k,i} \right)} \right] - \left( {1 - g\left( {k,i} \right)} \right)\log \left[ {1 - p\left( {k,i} \right)} \right]$$

where N represents the number of pixels, and $p\left( {k,i} \right) \in \left[ {0,1} \right]$ and $g\left( {k,i} \right) \in \left\{ {0,1} \right\}$ are, respectively, the predicted image and ground truth for class k.

Finally, we design a joint loss $L_{total}$ consisting of Dice loss $L_{Dice}$ and cross-entropy loss $L_{BCE}$ to perform all segmentation tasks. The formula is defined as follows:

10 $$L_{total} = \alpha L_{Dice} + \left( {1 - \alpha } \right)L_{BCE}$$

The weight $\alpha$ is set to 0.3 via experiments with different weights, which can obtain the best segmentation performance.

### Experimental setup

In this section, we first introduce the medical image datasets, experiment settings, and evaluation metrics in our experiment.

#### Dataset statement

In the experiment, our approach was evaluated on three publicly available medical image datasets and one clinical tongue image dataset. All the experiments were carried out in compliance with relevant guidelines and regulations. Informed consent was obtained from all participants and/or their legal guardians.

1. The tongue image segmentation task was to segment the tongue body from the TongeImageDataset^39^. The tongue dataset contains 300 images with their respective label images published by BioHit. The size of each tongue image is 768 × 576 pixels. These images have been resized to 512 × 512 pixels. These samples were randomly split into the training, validation, and test sets with a ratio of 8:1:1.
2. The public digital retinal images for vessel extraction (DRIVE) dataset came from a diabetic retinopathy screening program in the Netherlands^40^. It contains 40 images and their corresponding label images. The image dimension is 512 × 512 pixels. It can be freely downloaded from the official website. The 40 images were divided into 20 images for training and 20 images for testing. In addition, the 20 training images were randomly split into 16 for training and 4 for validation.
3. The two-dimensional (2D) CT lung images were obtained from the Lung Nodule Analysis (LUNA) competition^41^. We used this dataset to further evaluate the performance of the proposed MEA-Net. The challenge dataset contains 267 lung 2D images and their respective label images. The size of the images is 512 × 512 pixels. In the experiment, 267 2D samples were randomly divided into 213 training, 27 validation, and 27 test images.
4. The clinical tongue image dataset in this study was collected from the Shanghai University of Traditional Chinese Medicine, Shanghai, China. Informed consent to publish identifying images has been obtained. The tongue images were captured by specialized equipment in an open environment. The images were annotated by clinical experts. An additional problem is that images captured in an open environment are vulnerable to light intensity, complex backgrounds, and other factors that would make segmentation more difficult. There are 300 tongue images with a dimension of 1080 × 1440 in the original dataset but have been resized to 512 × 512 due to computational limitations. In our experiments, we used 80% of the dataset for training, whereas the remaining 20% were used for validation and testing.

#### Experiment settings

The implementation is based on the public PyTorch platform. The training and testing beds are Windows 10 systems with an NVIDIA GeForce RTX 2080 TI graphics card. During training, we used the Adam optimizer^42^ to train our network with batch size 4, with its hyperparameters set to the default values, where the initial learning rate lr = 2e−3, betas = (0.5, 0.999). The maximum epoch is 300.

Meanwhile, data augmentation was applied to avoid model overfitting including rotation, flip, translation, and mirroring. The images of all training datasets and their labels are used as input images into all methods. We also used five-fold cross-validation on four datasets. These results are shown in Tables 1, 2, 3, 4. The cross-validation approach was used to evaluate the performance of the network and obtain as much valid information as possible from the small dataset.

**Table 1:** Performance comparison on tongue segmentation (mean ± standard deviation).

Network	Accuracy	Sensitivity	Dice	AUC	BF-score
U-Net^1^	0.9954 ± 0.0029	0.9890 ± 0.0117	0.9886 ± 0.0066	0.9917 ± 0.0060	0.8817 ± 0.1017
Attention U-Net^16^	0.9953 ± 0.0045	0.9882 ± 0.0183	0.9884 ± 0.0093	0.9902 ± 0.0092	0.9013 ± 0.0882
R2U-Net^45^	0.9941 ± 0.0057	0.9786 ± 0.0244	0.9850 ± 0.0124	0.9814 ± 0.0121	0.7788 ± 0.1563
ResNet50^13^	0.9940 ± 0.0017	0.9850 ± 0.0094	0.9881 ± 0.0040	0.9906 ± 0.0044	0.8856 ± 0.1309
CE-Net^14^	0.9952 ± 0.0015	0.9897 ± 0.0067	0.9898 ± 0.0031	0.9879 ± 0.0032	0.8945 ± 0.1013
MultiResUNet^7^	0.9934 ± 0.0029	0.9805 ± 0.0142	0.9843 ± 0.0064	0.9909 ± 0.0069	0.8702 ± 0.1110
nnUnet^43^	0.9954 ± 0.0012	0.9874 ± 0.0046	**0.9903** ± 0.0037	0.9927 ± 0.0034	0.8101 ± 0.0836
MEA-Net (ours)	**0.9957** ± 0.0010	**0.9904** ± 0.0015	0.9902 ± 0.0022	**0.9938** ± 0.0010	**0.9075** ± **0.0841**

Significant values are in bold.

**Table 2:** Performance comparison on retinal vessel image segmentation (mean ± standard deviation).

Network	Accuracy	Sensitivity	Dice	AUC	BF-Score
U-Net^1^	0.9635 ± 0.0075	0.7638 ± 0.0496	0.8060 ± 0.0097	0.8433 ± 0.0238	0.6831 ± 0.0878
CE-Net^14^	0.9545 ± 0.0068	0.8125 ± 0.0443	0.8067 ± 0.0139	0.9005 ± 0.0214	0.6936 ± 0.1033
ET-Net^25^	0.9560 ± 0.0076	0.7893 ± 0.1257	0.8081 ± 0.0419	0.8988 ± 0.0582	0.7014 ± 0.1044
AEC-Net^26^	0.9674 ± 0.0087	0.8173 ± 0.0479	0.8288 ± 0.0242	0.8444 ± 0.0227	0.7027 ± 0.1137
AA-UNet^5^	0.9542 ± 0.0052	0.8079 ± 0.0576	0.8204 ± 0.0144	0.8907 ± 0.0271	0.6885 ± 0.0950
DGFAU-Net^19^	0.9577 ± 0.0065	0.7583 ± 0.0459	0.7576 ± 0.0084	0.8821 ± 0.0220	0.6972 ± 0.1035
CSAU^46^	0.9601 ± 0.0057	0.8229 ± 0.0419	0.8297 ± 0.0105	0.7281 ± 0.0201	0.6622 ± 0.1110
nnUnet^43^	0.9690 ± 0.0040	0.7873 ± 0.0364	0.8115 ± 0.0120	0.9109 ± 0.0246	0.8064 ± 0.1360
MEA-Net (ours)	**0.9736** ± 0.0064	**0.8349** ± 0.0594	**0.8377** ± 0.0131	**0.9113** ± 0.0282	**0.8987** ± 0.0216

Significant values are in bold.

**Table 3:** Performance comparison on lung segmentation (mean ± standard deviation).

Network	Accuracy	Sensitivity	Dice	AUC	BF-Score
U-Net^1^	0.9923 ± 0.0024	0.9824 ± 0.0078	0.9834 ± 0.0083	0.9818 ± 0.0031	0.9135 ± 0.0851
ET-Net^25^	0.9868 ± 0.0069	0.9765 ± 0.0104	0.9832 ± 0.0177	0.9911 ± 0.0053	0.9014 ± 0.0940
AEC-Net^26^	0.9927 ± 0.0019	0.9810 ± 0.0094	0.9843 ± 0.0071	0.9917 ± 0.0038	0.9083 ± 0.0890
CE-Net^14^	0.9935 ± 0.0019	0.9876 ± 0.0089	0.9852 ± 0.0057	0.9916 ± 0.0038	0.9208 ± 0.0970
Attention U-Net^16^	0.9922 ± 0.0023	0.9765 ± 0.0112	0.9832 ± 0.0067	0.9908 ± 0.0052	0.9197 ± 0.0848
CPFNet^18^	0.9895 ± 0.0022	0.9837 ± 0.0083	0.9843 ± 0.0071	0.9907 ± 0.0032	0.9129 ± 0.0466
MultiResUNet^7^	0.9932 ± 0.0024	0.9903 ± 0.0085	0.9829 ± 0.0071	0.9922 ± 0.0035	0.9183 ± 0.0455
nnUnet^43^	0.9937 ± 0.0028	**0.9907** ± 0.0054	0.9823 ± 0.0078	0.9922 ± 0.0045	0.9164 ± 0.0450
MEA-Net (ours)	**0.9942** ± 0.0022	0.9903 ± 0.0103	**0.9858** ± 0.0057	**0.9923** ± 0.0046	**0.9332** ± 0.0362

Significant values are in bold.

**Table 4:** Performance comparison on clinical tongue image segmentation (mean ± standard deviation).

Network	Accuracy	Sensitivity	Dice	AUC	BF-Score
U-Net^1^	0.9985 ± 0.0024	0.8836 ± 0.2339	0.9025 ± 0.2010	0.7913 ± 0.1169	0.8969 ± 0.1799
CE-Net^14^	0.9987 ± 0.0011	0.9356 ± 0.1711	0.9231 ± 0.1681	0.8823 ± 0.0855	0.9372 ± 0.0820
MutiResUNet^7^	0.9984 ± 0.0022	0.9147 ± 0.1825	0.9183 ± 0.1386	0.8818 ± 0.0912	0.8893 ± 0.1593
Attention U-Net^16^	0.9983 ± 0.0029	0.8791 ± 0.2533	0.8862 ± 0.2170	0.8773 ± 0.1266	0.8603 ± 0.2204
ResNet50^13^	0.9990 ± 0.0005	0.9417 ± 0.0644	0.9547 ± 0.0375	0.8659 ± 0.0322	0.9260 ± 0.1028
nnUnet^43^	0.9993 ± 0.0005	0.9687 ± 0.0275	0.9678 ± 0.0198	0.9833 ± 0.0151	0.8783 ± 0.1554
MEA-Net (ours)	**0.9993** ± 0.0004	**0.9701** ± 0.0208	**0.9704** ± 0.0141	**0.9849** ± 0.0104	**0.9521** ± 0.0657

Significant values are in bold.

#### Methods for comparison

Several comparison methods were selected for application to four datasets, such as U-Net^1^, MutiResUNet^7^, ResNet50^13^, CE-Net^14^, Attention U-Net^16^, and nnUnet^43^. Meanwhile, some of the comparison methods were applied to specific datasets. For example, ET-Net^25^ and AEC-Net^26^ were applied to the DRIVE and LUNA datasets. All comparison experiments were carried out by the above hardware equipment with the parameter settings of the relevant papers.

#### Evaluation metrics

To evaluate segmentation performance, we used accuracy (Acc), sensitivity (Sen), and the Dice coefficient (Dice) to measure the accuracy of semantic segmentation for medical images, which are, respectively, defined as follows Eqs. (11)–(13). Besides, BF-Score is calculated to decide whether a boundary point has a match or not^44^, which is defined as Eq. (14):

11 $$Accuracy = \frac{{\sum\nolimits_{i = 1}^{N} {\frac{TP + TN}{{TP + TN + FP + FN}}} }}{N}$$

12 $$Sensitivity = \frac{{\sum\nolimits_{i = 1}^{N} {\frac{TP}{{TP + FN}}} }}{N}$$

13 $$Dice = \frac{{\sum\nolimits_{i = 1}^{N} {\frac{2 \times TP}{{2 \times TP + FP + FN}}} }}{N}$$

14 $$BF-Score = \frac{{\sum\nolimits_{i = 1}^{N} {\frac{{2 \times \frac{TP}{{TP + FP}} \times \frac{TP}{{TP + FN}}}}{{\frac{TP}{{TP + FP}} + \frac{TP}{{TP + FN}}}}} }}{N}$$

where TP, FP, TN, and FN denote true positives, false positives, true negatives, and false negatives, respectively. N is the total number of test images.

The area under the receiver operating characteristic curve (AUC) was used to evaluate the performance of the models. The AUC will be equal to 1 when the model is perfect.

## Results

### Tongue image segmentation

We compared the proposed MEA-Net with existing state-of-the-art algorithms, including U-Net^1^, Attention U-Net^16^, R2U-Net^45^, ResNet50^13^, CE-Net^14^, MultiResUNet^7^, and nnUnet^43^. As shown in Table 1, our proposed MEA-Net achieved 0.9957, 0.9904, and 0.9902 in terms of Acc, Sen, and Dice. Compared with MultiResUNet, the Acc, Sen, and Dice of the proposed method increased by 0.0023, 0.0099, and 0.0059, respectively. Furthermore, the AUC of the proposed network reached 0.9938.

**Figure 6:**
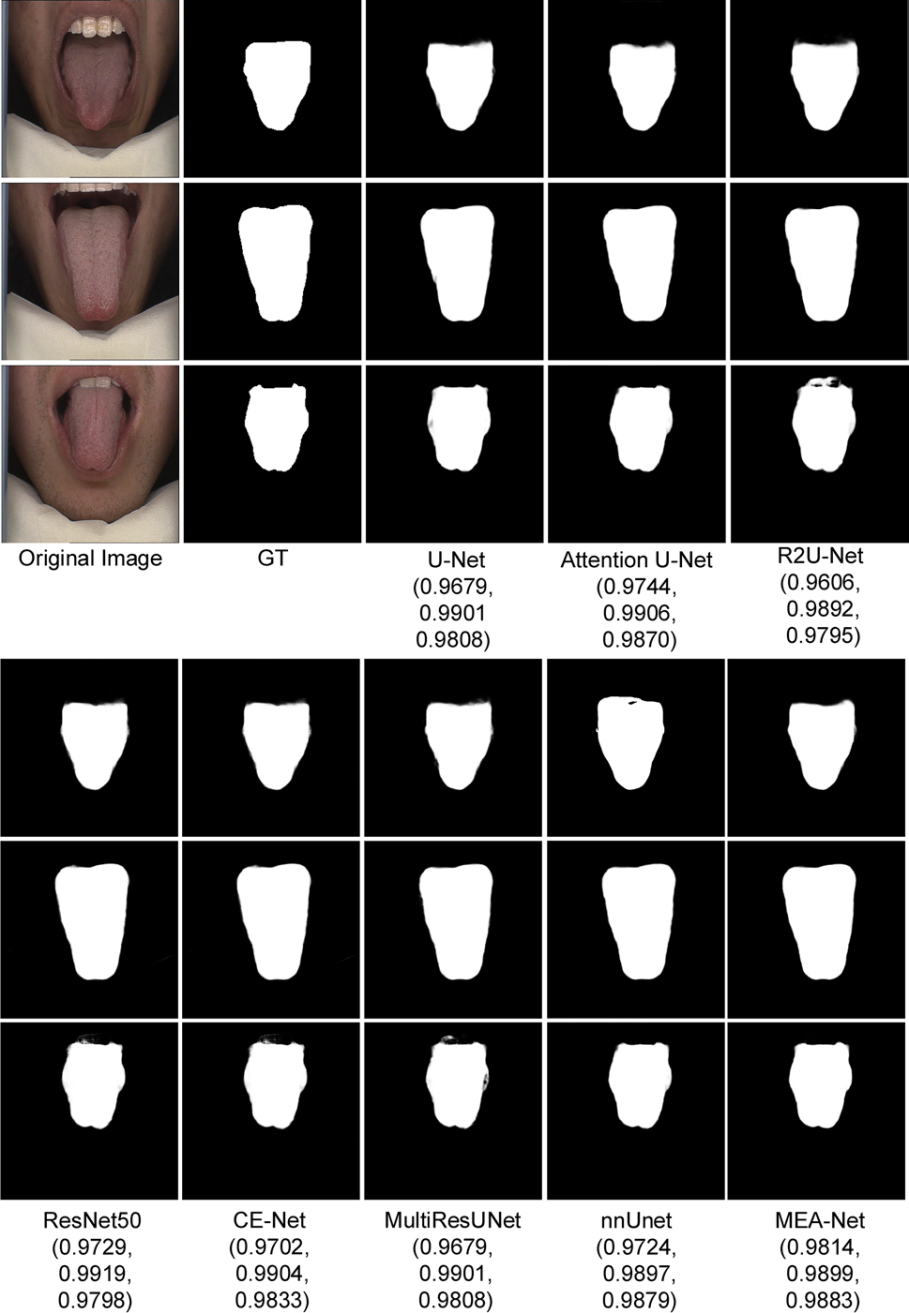
Sample results of tongue image segmentation. (The Dice values for each legend are in brackets).

As can be seen from Table 1, the above metrics of nnUnet were the same as those of our proposed MEA-Net. Although the difference in Dice values between the two networks was 0.001, the standard deviation in MEA-Net was smaller. The BF-Score of our proposed MEA-Net reached 0.9075, which was 0.0974 higher than that of nnUnet. The performances of these methods are similar to that of the proposed network because the tongue images acquired in the controlled environment only contain the mouth area and part of the face area. The DL-based networks can better eliminate irrelevant areas (lips and teeth) with an Acc greater than 0.9. Figure 6 shows examples of tongue image segmentation for visual comparison. Each testing image has its corresponding Dice value in Fig. 6. (The subsequent figures are shown in the same way.) The visual comparisons are very close, so the Dice values of all compared methods for each example image further show the superiority of the proposed method.

### Retinal vessel image segmentation

**Figure 7:**
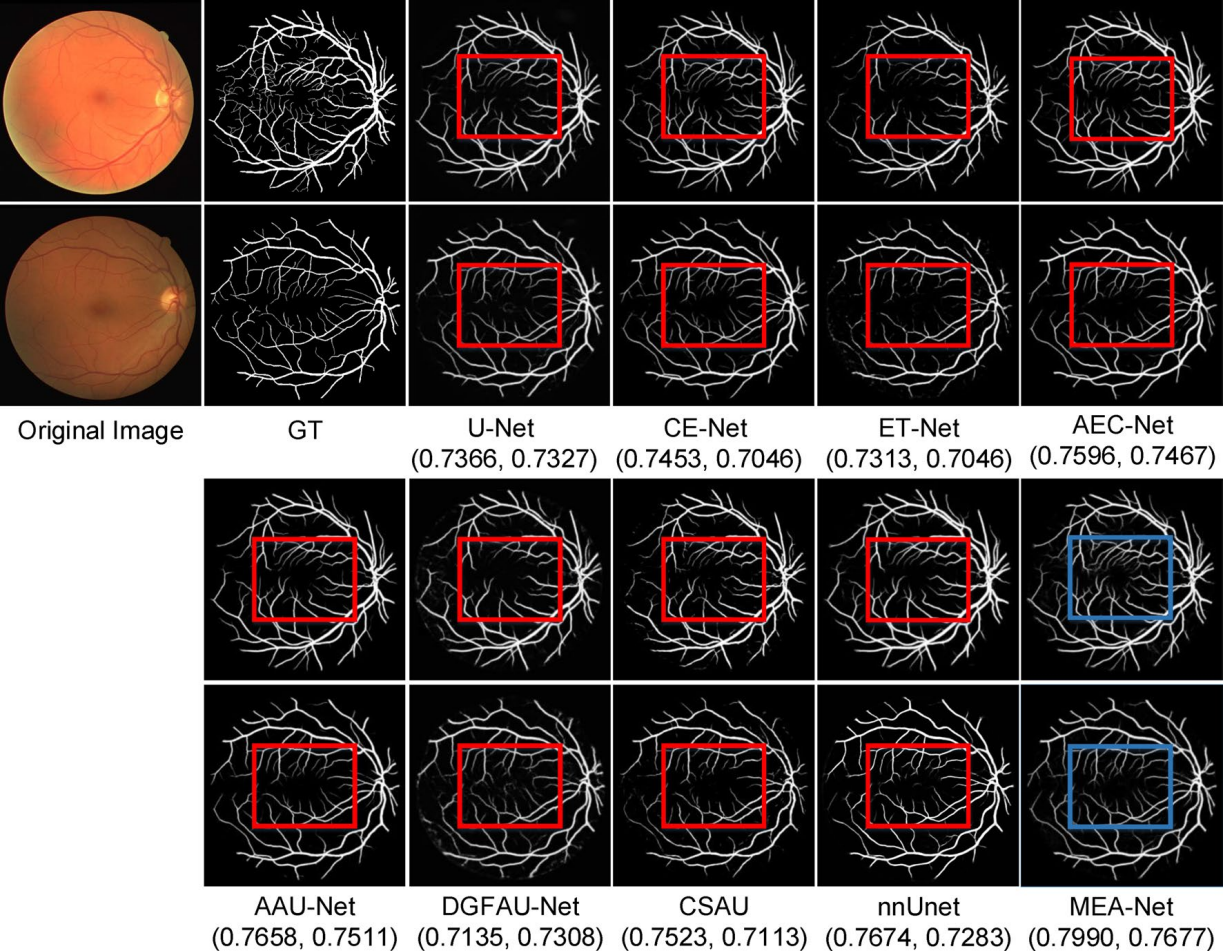
Sample results of DRIVE segmentation. (The Dice values for each legend are in brackets).

We compared the proposed MEA-Net with state-of-the-art algorithms, including U-Net^1^, CE-Net^14^, ET-Net^25^, AEC-Net^26^, AA-UNet^5^, DGFAU-Net^19^, CSAU^46^, and nnUnet^43^. As shown in Table 2, our proposed MEA-Net achieved 0.9736, 0.8349, 0.8377, and 0.9113 in terms of Acc, Sen, Dice, and AUC, respectively, which were superior to those of other methods. The Dice and AUC of the proposed network were 0.0317 and 0.0680—higher than those of the classical U-Net, which implied that MEA-Net was suitable for retinal vessel segmentation. Compared to other comparison networks, nnUnet showed substantial improvements in the results, especially the BF-Score value. Our method achieved 0.8987 in BF-Score, which was 0.0823 higher than that of nnUnet. Our method, ET-Net, and AEC-Net are all edge attention networks, but the results of our methods show an improvement over previous methods. Some examples for visual comparison are shown in Fig. 7, which show that more detailed blood vessels can be segmented and their edges are clearer in blue rectangles via MEA-Net. The Dice values were given below for each comparison method.

### Lung image segmentation

**Figure 8:**
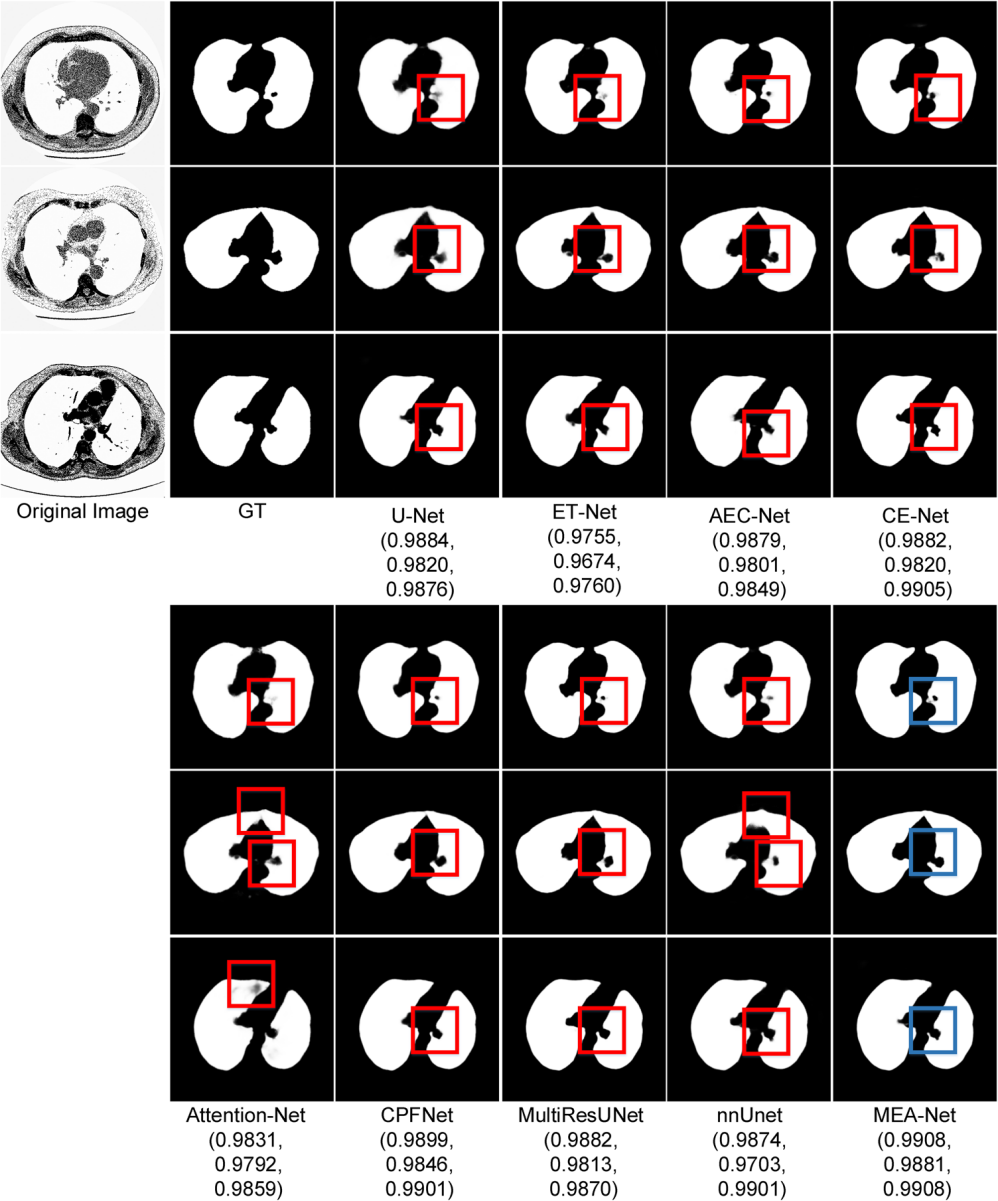
Sample results of LUNA segmentation. (The Dice values for each legend are in brackets).

We compared our method with other excellent encoder–decoder structures, including U-Net^1^, ET-Net^25^, AEC-Net^26^, CE-Net^14^, Attention U-Net^16^, CPFNet^18^, MultiResUNet^7^, and nnUnet^43^. From the comparison shown in Table 3, the MEA-Net achieved 0.9942 in Acc, 0.9903 in Sen, and 0.9858 in Dice, which was better than U-Net. In comparison to the performances of ET-Net, Acc increased from 0.9868 to 0.9942, Sen increased from 0.9765 to 0.9903, and Dice increased from 0.9832 to 0.9858 by 0.0026. In addition, the MEA-Net achieved 0.9923 in AUC which was higher than other methods, and proved that the new encoder–decoder structure with the edge module was beneficial for lung segmentation as well. The MEA-Net reached 0.9332 in BF-Score which was 0.0168 higher than that of nnUnet. Figure 8 shows some examples for visual comparison. It can be seen that it is difficult for the lung image segmentation task to segment details (in the red rectangles) in the lung. The proposed MEA-Net can use the edge module to detect the circle and restore the edge information (in the blue rectangles).

### Clinical image segmentation

**Figure 9:**
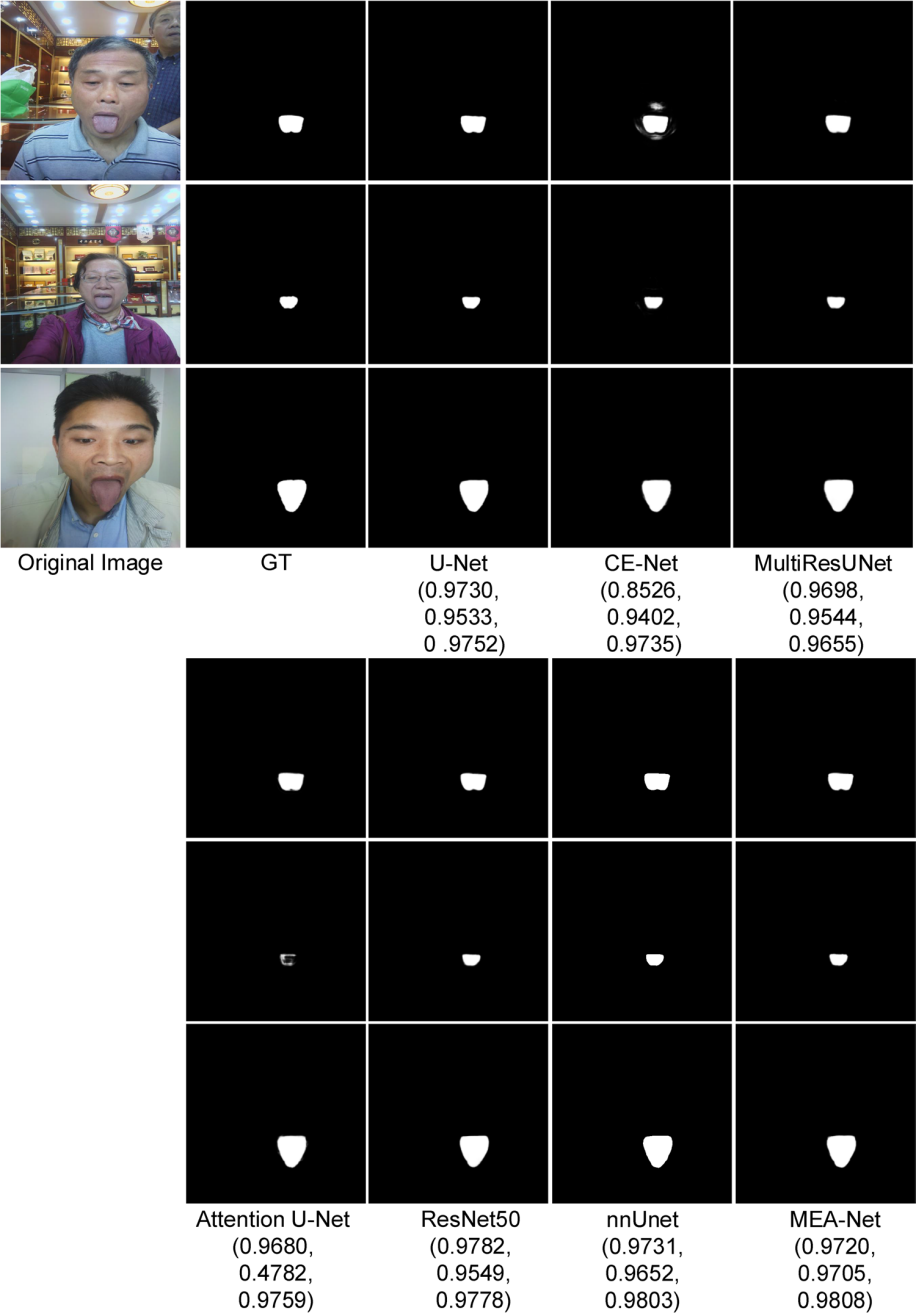
Sample results of clinical image segmentation. (The Dice values for each legend are in brackets).

We compared the proposed MEA-Net with state-of-the-art algorithms, including U-Net^1^, CE-Net^14^, MultiResUNet^7^, Attention U-Net^16^, ResNet50^13^, and nnUnet^43^. As shown in Table 4, our proposed MEA-Net achieved 0.9993, 0.9701, 0.9704, and 0.9849 in terms of Acc, Sen, Dice, and AUC respectively. Compared to the performances of MultiResUNet, our method’s Acc increased from 0.9984 to 0.9993, its Sen increased from 0.9147 to 0.9701, and its Dice increaseds from 0.9183 to 0.9704 by 0.0521. Compared to U-Net, the proposed network had a great improvement in AUC and BF-Score, which increased by 0.1936 and 0.0552. The proposed MEA-Net can still obtain satisfactory performances in an open environment. Some examples for visual comparison of clinical tongue image segmentation were shown in Fig. 9. The figure shows that MEA-Net has more detailed edge information than previous networks. For example, CE-Net not only produces disconnected lip areas but also loses the edge information of the tongue region. This may cause clinical experts to make incorrect image diagnoses.

### Ablation studies

To further evaluate the effectiveness of MEA-Net, we conducted ablation studies using four different datasets as examples. The results were listed in Table 5. In the ablation studies, we used ResNet50 instead of the feature encoder as the backbone and chose the encoder–decoder structure shown in Fig. 1 as the baseline. In Table 5, the performance of the proposed MEA-Net was higher than those of the other combinations.

**Table 5:** Ablation studies for the edge module on four datasets (mean ± standard deviation).

Network	Tongue	DRIVE	LUNA	Clinical
	Dice			
U-Net	0.9886 ± 0.0066	0.8060 ± 0.0097	0.9834 ± 0.0083	0.9025 ± 0.2010
U-Net + Edge Module	0.9899 ± 0.0205	0.8172 ± 0.0090	0.9828 ± 0.0126	0.9101 ± 0.0637
Backbone	0.9850 ± 0.0153	0.8306 ± 0.0835	0.9814 ± 0.0121	0.9547 ± 0.0375
Backbone + Edge Module	0.9885 ± 0.0139	0.8321 ± 0.0841	0.9852 ± 0.0053	0.9552 ± 0.0274
Baseline	0.9865 ± 0.0156	0.8331 ± 0.0516	0.9852 ± 0.0064	0.9439 ± 0.0540
Baseline + Edge Module (without MAG)	0.9885 ± 0.0219	0.8359 ± 0.0131	0.9857 ± 0.0096	0.9457 ± 0.0303
Baseline + Edge Module (without EFE)	0.9887 ± 0.0163	0.8330 ± 0.0513	0.9852 ± 0.0063	0.9472 ± 0.0307
Baseline + Edge Module (E1)	0.9858 ± 0.0398	0.8206 ± 0.0500	0.9811 ± 0.0098	0.9583 ± 0.0483
Baseline + Edge Module (E1 + E2 + E3)	0.9854 ± 0.0158	0.8296 ± 0.0091	0.9884 ± 0.0087	0.9523 ± 0.0165
Baseline + Edge Module (E1 + E2 + E3 + E4)	0.9842 ± 0.0215	0.8028 ± 0.0144	0.9850 ± 0.0061	0.9600 ± 0.0167
Baseline + Edge Module (E2)	0.9854 ± 0.0145	0.8050 ± 0.0152	0.9844 ± 0.0061	0.9648 ± 0.0194
Baseline + Edge Module (E2 + E3)	0.9878 ± 0.0099	0.8029 ± 0.0288	0.9842 ± 0.0068	0.9640 ± 0.0203
Baseline + Edge Module (E2 + E3 + E4)	0.9858 ± 0.0116	0.7913 ± 0.0140	0.9732 ± 0.0183	0.9602 ± 0.0149
Baseline + Edge Module (E3)	0.9882 ± 0.0092	0.7988 ± 0.0090	0.9791 ± 0.0113	0.9626 ± 0.0170
Baseline + Edge Module (E4)	0.9833 ± 0.0169	0.8029 ± 0.0104	0.9847 ± 0.0053	0.9549 ± 0.0311
Baseline + Edge Module (E3 + E4)	0.9806 ± 0.0231	0.7921 ± 0.0162	0.9805 ± 0.0081	0.9626 ± 0.0359
MEA-Net (E1 + E2)	**0.9902** ± 0.0022	**0.8377** ± 0.0131	**0.9885** ± 0.0057	**0.9704 ± 0.0141**

Significant values are in bold.

As shown in Table 5, the baseline achieved Dice values of 0.9865, 0.8331, 0.9852, and 0.9439 on TongueImageDataset, DRIVE, LUNA, and clinical images, respectively. In DRIVE and LUNA datasets, the Dice value of the proposed encoder–decoder structure was higher than that of U-Net and Backbone. Furthermore, when we appended the proposed edge module to the backbone (backbone + edge module), the performance in different datasets was slightly improved. It is demonstrated that both the new encoder–decoder structure and the edge module are beneficial for medical image segmentation in these datasets. ResNet50 with the edge module had a small improvement in terms of Dice, but the result was lower than that of the proposed network. These results indicate that pre-trained ResNet50 blocks are unsuitable for these medical image datasets.

To study the effect of EFE, we only added EFE to the baseline (baseline + EFE), and the results prove that the edge module could guide the network to learn edge information that is important for segmentation. In addition, we appended the MAG module to the baseline without the EFE module (baseline + MAG). For example, compared with the baseline network, the Dice value in TongueImageDataset increased from 0.9865 to 0.9887 by 0.0022, demonstrating that the MAG module has the learning capability to choose the edge information for the segmentation task.

We also conducted an ablation study for the EFE module. Different encoding blocks (including E1, E2 E3, and E4) were combined in comparative experiments. After a series of convolution upsampling operations, the output size of each compared EFE module was restored to the same size as that of E1. The EFE module produced feature maps with different channel information. Each feature map was then multiplied by E1 to produce new features with attentional bias, thus the output size of the EFE was the same as that of E1.

The proposed EFE module used different encoding stages to produce edge attention maps. First, we tested the EFE module with E1 (baseline + edge module (E1)) in four different datasets but the performance was not better than that of the proposed baseline. Next, we tested the EFE module with E1 and E2 (MEA-Net (E1 + E2)). The comparison results showed that our MEA-Net reached better results in four datasets. It can be observed that this combination can use the edge information in the early stages to produce useful attention maps. In addition, we tested the EFE module with three encoding stages (baseline + edge module (E1 + E2 + E3)). In the DRIVE dataset, compared to MEA-Net, the Dice value decreased from 0.8377 to 0.8296 by 0.0081. The network may have redundant information even though the edge guidance maps are produced from three encoding stages. This shows that after E3 passes through two pooling layers, it loses several low-level features, preventing it from acting as the edge guidance feature in the decoding path. As the number of encoding blocks increases, the segmentation performance of the network does not improve but rather decreases.

Particularly when using an encoding block alone (like Edge Module (E3) and Edge Module (E4)), the performance of the segmentation was significantly reduced. For example, the output size of E3 and E4 became very small in the encoding process. The directly upsampling operation to recover to the same size as E1 loses a lot of information. Meanwhile, a lack of rich edge information will be detrimental to the subsequent guided assignment of weights by the MAG module.

### Discussion

In this section, we discuss the performance of the proposed network compared to other networks in different medical image segmentation tasks. To capture and use the edge information in the encoding path and obtain a better performance in medical segmentation tasks, we proposed a new encoder–decoder structure with an edge module called MEA-Net. The edge module consists of EFE and MAG modules. The main focuses of the proposed network are as follows: (1) Design a new feature encoder to replace the pretrained backbone of ResNet50 to extract more information that better matches the characteristics of medical images. (2) Design a new feature decoder by skip connection and attention mechanism to fuse the various information between the encoding and decoding paths. (3) Propose the EFE and MAG module in the edge branch to obtain more detailed edge information and eliminate redundant information. (4) Test MEA-Net on four different medical datasets.

Previous state-of-the-art networks for medical image segmentation focused on how to use larger receptive fields to improve the ability to capture multiscale information. However, these networks ignore low-level features. Our proposed network focused on making full use of edge information, which is a low-level feature. We used BF-Score as quantitative results of the edge segmentation. In the DRIVE database, the proposed network showed an improvement in BF-Score, as can detect and segment the detailed edges of the retinal vessel. As shown in Tables 1 and 3, compared to other networks, the proposed MEA-Net improved the edge result as shown in higher BF-Score. As shown in Fig. 8, some details in the lung were able to be detected and segmented. Because of the edge module, the network, during training, was able to obtain and send the circle information to the decoding path. In addition, the proposed network achieved excellent performance in clinical image segmentation, as shown in Table 4. Although images were taken in an open environment, the edge module was able to filter irrelevant edge information so that the network can detect the segmentation region.

To further evaluate the effectiveness and robustness of MEA-Net, we performed several ablation experiments, as shown in Table 5. The new encoder–decoder structure as the baseline showed to be more suitable than U-Net and the backbone. As U-Net only uses two common 3 × 3 convolutions to capture features, it is difficult to discover more information. ResNet50 applied the residual connection to deepen the network, but it was not beneficial for medical image segmentation. Table 5 shows that the performances of U-Net and the backbone of ResNet50 are weaker than the proposed feature encoder and decoder. In addition, we designed different combinations of the three models to validate the efficacy of the edge module. These Dice values have been slightly improved. This reveals that the proposed EFE and MAG modules can choose effective edge features and improve the performance of the network. The MAG module uses the characteristics of each attention map to obtain different edge information.

As shown in Table 5, the combination of E1 and E2 in the EFE module is the best option because E2 contains necessary edge information, and inversely, E3 and E4 have small-size high-dimensional information; thus, redundant information can be easily produced during the upsampling operation. Experimental results demonstrate that the new encoder–decoder structure with the edge module in E1 and E2 uses edge information for segmentation tasks. This can explain why the proposed MEA-Net is more beneficial for medical image segmentation.

Even though the proposed network has achieved good results in different segmentation tasks, it still has some limitations: (1) The network concentrates on edge information and ignores high-level features in the encoding and decoding paths. (2) Our model is designed for 2D medical image segmentation. In recent years, three-dimensional (3D) medical applications have become increasingly desirable for various medical image segmentation tasks. (3) Compared with the other three datasets, the DRIVE dataset contains a relatively small number of images even though data augmentation can be applied to it. In our future work, we aim to use both low-level and high-level features based on the components of MAE-Net in 3D medical image segmentation^43^.

In conclusion, our experimental results indicate that the developed MEA-Net can combine multilayer edge information in different encoding paths, which can improve segmentation performance in different tasks.

## Supplementary Information

Supplementary Information.
