# Supplementary material for: MEA-Net: multilayer edge attention network for medical image segmentation
Source: Sci Rep. 2022 May 12;12:7868. doi: 10.1038/s41598-022-11852-y (PMC9098486; doi:10.1038/s41598-022-11852-y)
Supplement: Supplementary file 1 — Supplementary Information. [file 41598_2022_11852_MOESM1_ESM.pdf]

MEA-Net: Multilayer Edge Attention Network for Medical Image Segmentation

Huilin Liu[1], Yue Feng[1,*], Hong Xu[1,2], Shufen Liang[1], Huizhu Liang[1], Shengke Li[1], Jiajian Zhu[1], Shuai Yang[3] & Fufeng Li[3,*]

[1] Faculty of Intelligent Manufacturing, Wuyi University, Jiangmen, Guangdong, China

[2] Victoria University, Melbourne, Australia

[3] Laboratory of TCM Four Processing, Shanghai University of TCM, Shanghai, China

[*] Corresponding author E-mail:

yfeng_wyu@wyu.edu.cn

li_fufeng@aliyun.com

Appendix

We tested the proposed baseline with or without decoder 4. As we can see from the Table S1, the experimental results of the baseline structure with the decoder 4 are not satisfactory, especially in the DRIVE dataset, where the results drop substantially. Adding a decoder requires additionally processing the feature information from the encoding path. Moreover, with the addition of D4, the convolution-pooling operation makes the image size smaller, which does not facilitate the decoding path to recover image information. Therefore, this network structure did not add D4.

Table S1 Ablation studies for baseline on four datasets (mean ± standard deviation)

Dataset	Network	Accuracy	Sensitivity	Dice	AUC	BF-Score
Tongue	Baseline + D4	0.9952±0.0008	0.9857±0.0023	0.9881±0.0018	0.9937±0.0012	0.9038±0.0171
	Baseline	0.9943±0.0059	0.9821±0.0262	0.9865±0.0156	0.9890±0.0128	0.8922±0.0946
Drive	Baseline + D4	0.9682±0.0071	0.7674±0.0429	0.7979±0.0138	0.8602±0.0210	0.6704±0.1014
	Baseline	0.9725±0.0059	0.8289±0.0460	0.8331±0.0516	0.8773±0.0216	0.7056±0.0818
Lung	Baseline + D4	0.9919±0.0023	0.9777±0.0101	0.9826±0.0072	0.9911±0.0043	0.9099±0.0905
	Baseline	0.9942±0.0020	0.9881±0.0096	0.9852±0.0064	0.9920±0.0042	0.9168±0.0661
Clinical	Baseline + D4	0.9985±0.0031	0.9028±0.2339	0.9067±0.2177	0.9512±0.1169	0.8959±0.1872
	Baseline	0.9992±0.0004	0.9416±0.0240	0.9439±0.0540	0.9764±0.0120	0.9582 0.0648

We experimented with E1 and E2 as an input to the MAG as well. As can be seen from the Table S2, When E2 is also used as an input to the MAG, their segmentation performance for four datasets does not improve, but rather decreases, because it actually causes redundancy of information. E2 needs to be set to the same size and number of channels as E1, and then multiplied with each single attention map for attention weights. The Experimental results show that using E1 alone as the input of MAG obtains better segmentation performance.

Table S2 Ablation studies for the input of MAG in MEA-Net on four datasets (mean ± standard deviation)

Dataset	The input of MAG	Accuracy	Sensitivity	Dice	AUC	BF-Score
Tongue	E1+E2	0.9940±0.0068	0.9821±0.0284	0.9859±0.0149	0.9898±0.0139	0.8975±0.1173
	E1	0.9957±0.0010	0.9904±0.0015	0.9902±0.0022	0.9938±0.0010	0.9075±0.0841
Drive	E1+E2	0.9698±0.0066	0.7558±0.0763	0.7948±0.0096	0.8723±0.0358	0.6822±0.0945
	E1	0.9736±0.0064	0.8349±0.0594	0.8377±0.0131	0.9113±0.0282	0.8064±0.1360
Lung	E1+E2	0.9941±0.0017	0.9899±0.0087	0.9852±0.0056	0.9926±0.0037	0.9244±0.0387
	E1	0.9942±0.0022	0.9903±0.0103	0.9858±0.0057	0.9923±0.0046	0.9332±0.0362
Clinical	E1+E2	0.9993±0.0004	0.9689±0.0432	0.9672±0.0238	0.9842±0.0216	0.9418±0.1037
	E1	0.9993±0.0004	0.9701±0.0208	0.9704±0.0141	0.9849±0.0104	0.9521±0.0657

We conducted an experimental comparison of the two loss functions as shown in Table S3. Loss function 1 is adding dice loss for the final loss, while loss function 2 is the designed loss function in Loss Function Section. Both loss function designs are relatively close, but the experimental results of our proposed loss function are slightly improved, so we continue with the original loss function design.

Table S3 Ablation studies for loss function in MEA-Net on four datasets (mean ± standard deviation)

Dataset	Loss function	Accuracy	Sensitivity	Dice	AUC	BF-Score
Tongue	Loss1	0.9925±0.0119	0.9760±0.0489	0.9822±0.0277	0.9867±0.0242	0.8922±0.1147
	Loss2	0.9957±0.0010	0.9904±0.0015	0.9902±0.0022	0.9938±0.0010	0.9075±0.0841
Drive	Loss1	0.9717±0.0056	0.8289 0.0377	0.8190 0.0105	0.9066±0.0184	0.6283±0.1242
	Loss2	0.9736±0.0064	0.8349±0.0594	0.8377±0.0131	0.9113±0.0282	0.8064±0.1360
Lung	Loss1	0.9944±0.0018	0.9875 0.0095	0.9859 0.0054	0.9918±0.0042	0.9197±0.0548
	Loss2	0.9942±0.0022	0.9903±0.0103	0.9858±0.0057	0.9923±0.0046	0.9332±0.0362
Clinical	Loss1	0.9993±0.0004	0.9681±0.0279	0.9675±0.0175	0.9838±0.0139	0.9054 0.0920
	Loss2	0.9993±0.0004	0.9701±0.0208	0.9704±0.0141	0.9849±0.0104	0.9521±0.0657
